# Diagnosis of extrapulmonary tuberculosis using the MPT64 antigen detection test in a high-income low tuberculosis prevalence setting

**DOI:** 10.1186/s12879-020-4852-z

**Published:** 2020-02-12

**Authors:** Ida Marie Hoel, Lisbet Sviland, Heidi Syre, Anne Ma Dyrhol-Riise, Ingerid Skarstein, Peter Jebsen, Melissa Davidsen Jørstad, Harald Wiker, Tehmina Mustafa

**Affiliations:** 1 Centre for International Health, Department of Global Public Health and Primary Care, University of Bergen, Bergen, Norway; 2 Department of Clinical Science, University of Bergen, Bergen, Norway; 3 Department of Clinical Medicine, University of Bergen, Bergen, Norway; 4 Department of Pathology, Haukeland University Hospital, Oslo, Norway; 5 Department of Medical Microbiology, Stavanger University Hospital, Stavanger, Norway; 6 Department of Infectious Diseases, Oslo University Hospital, Oslo, Norway; 7 Institute of Clinical Medicine, University of Oslo, Oslo, Norway; 8 Department of Microbiology, Haukeland University Hospital, Bergen, Norway; 9 Department of Pathology, Oslo University Hospital, Oslo, Norway; 10 Department of Thoracic Medicine, Haukeland University Hospital, Bergen, Norway

**Keywords:** Extrapulmonary tuberculosis, Diagnostic test, Immunohistochemistry, MPT64, Antigen detection test

## Abstract

**Background:**

Extrapulmonary tuberculosis (EPTB) poses diagnostic challenges due to the paucibacillary nature of the disease. The immunochemistry-based MPT64 antigen detection test (MPT64 test) has shown promising results for diagnosing EPTB in previous studies performed in low-resource settings, with higher sensitivity than microscopy and culture. The aim of this study was to investigate the performance of the MPT64 test in a routine clinical setting in a high-income low TB prevalence country.

**Methods:**

Extrapulmonary samples sent for TB diagnostics to microbiology and pathology laboratories at three regional tertiary care hospitals in Norway in a one-year period were included and subjected to the MPT64 test in parallel to the routine TB diagnostic tests.

**Results:**

Samples from 288 patients were included and categorised as confirmed TB cases (*n* = 26), clinically diagnosed TB cases (*n* = 5), non-TB cases (*n* = 243) and uncategorised (*n* = 14), using a composite reference standard (CRS). In formalin-fixed biopsies, the sensitivity (95% CI) of the MPT64 test, microscopy, PCR-based tests pooled, and culture was 37% (16–62), 20% (4–48), 37% (16–62) and 50% (23–77), respectively, against the CRS. The MPT64 test showed a good positive predictive value (88%) and an excellent specificity (99, 95% CI 92–100) in formalin-fixed biopsies. In fine-needle aspirates, pus and fluid samples, the test performance was lower.

**Conclusions:**

The MPT64 test was implementable in pathology laboratories as part of routine diagnostics, and although the sensitivity of the MPT64 test was not better than culture in this setting, the test supplements other rapid diagnostic methods, including microscopy and PCR-based tests, and can contribute to strengthen the diagnosis of EPTB in formalin-fixed biopsies in the absence of culture confirmation.

## Background

While tuberculosis (TB) remains a global health problem, the incidence in Norway and many other high-income countries is low [1]. Still, diagnosis and control of TB disease poses significant challenges in high-income settings. Although TB rates have been continuously declining in the Norwegian-born population since the middle of the past century, the overall TB incidence in Norway and other high-income countries has remained relatively stable over the last years because of immigration from TB prevalent countries [2–5]. Several studies also report that the increase in foreign-born TB cases is associated with a rise in the proportion of extrapulmonary TB (EPTB) [3, 5–7]. In the European region, EPTB has increased from 16,4% of all TB cases in 2002 to 22,8% in 2016 [6, 8]. In the Netherlands, England, Australia and Norway, EPTB currently accounts for as much as 40% of all TB cases [1, 2].

The diagnosis of EPTB is challenging. Clinical and radiological findings are often non-specific and the sensitivity of routine TB diagnostic tests, including microscopy for acid fast bacilli (AFB) and culture, is low in paucibacillary disease [9]. Culture also requires advanced laboratory facilities, and results could be delayed up to 8 weeks. Globally, the use of rapid molecular tests for detection of TB is increasing, albeit most commercially available PCR-based tests are only approved for pulmonary TB. The only World Health Organization (WHO) endorsed PCR-based test for diagnosing EPTB, Xpert MTB/RIF (Cepheid, Sunnyvale, CA), has shown variable sensitivity in extrapulmonary samples [10] and is only recommended for subgroups of EPTB [11]. A recently launched new version, Xpert MTB/RIF Ultra (Xpert Ultra), performs better in smear negative, culture positive sputum samples [12], but so far, few studies have investigated its use in EPTB [13–18]. Histopathological findings suggestive of TB may support the EPTB diagnosis, but these are also present in other diseases including sarcoidosis and non-tuberculosis mycobacteria (NTM) infections. The incidence of NTM infections is also increasing in western countries [19–22]. Due to these diagnostic challenges, a definite diagnosis of EPTB is often difficult to obtain. Many EPTB patients are diagnosed clinically and EPTB is associated with diagnostic delay [23–25]. Thus, better diagnostic tests are needed to improve early case detection and management of EPTB patients.

An immunochemistry-based test for detection of the mycobacterial secreted protein MPT64 (MPT64 test) from biopsies, fine-needle aspirates (FNAs) and fluid samples has shown high sensitivity for diagnosing EPTB in previous studies compared to culture and a TB specific nested-PCR [26–31]. The MPT64 test is robust and fast, and can differentiate between NTM and TB disease, as the MPT64 protein is specific for *Mycobacterium tuberculosis* complex (MTBC) species, and not found in NTM [32–34]. A recent study conducted in Zanzibar, Tanzania, has also shown that the MPT64 test is implementable in a routine TB diagnostic setting in a TB high-endemic low-resource country [35]. However, the performance of the MPT64 test has not yet been evaluated in a routine clinical setting in a low TB burden high-income country. The objective of the study was to evaluate performance of the MPT64 test and whether the test would provide an added value to EPTB diagnostics when implemented in routine TB diagnostics in the high-resource health care system in Norway.

## Methods

### Sample inclusion

**Fig. 1:**
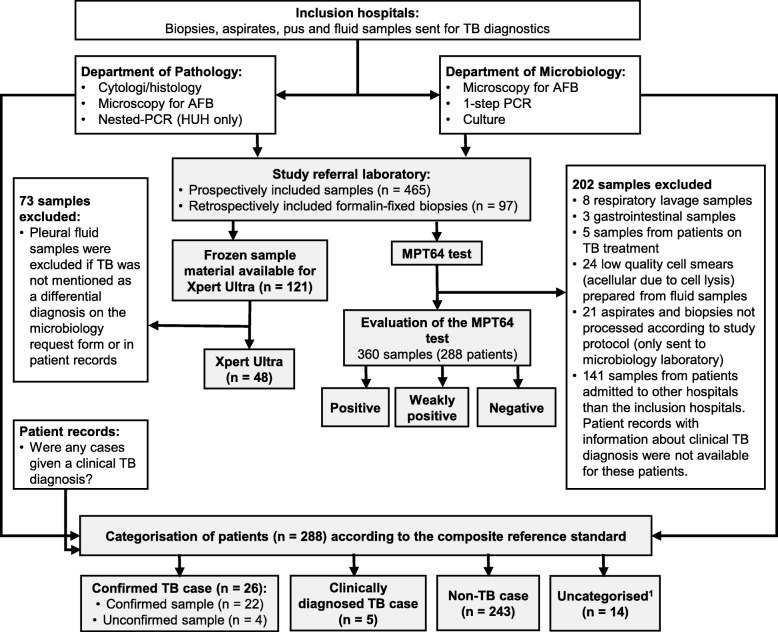
Flow chart of study design and sample inclusion. Abbreviations: TB, tuberculosis; PCR, polymerase chain reaction; AFB, acid fast bacilli. ^1^Uncategorised cases included 3 patients who died, 2 patients that did not show for clinical controls, 8 patients that had not been given a definite diagnosis 8 months after inclusion of samples ended, but for whom TB could not be ruled out either because they have previously been treated for TB or because histopathology showed necrotising granulomas and giant cells in the samples included. The last patient was given a clinical TB diagnosis, but the sample included in the study showed no pathology and may be non-representative of the site of infection

Formalin-fixed biopsies, FNAs and fluid samples sent for TB diagnostics to microbiology and pathology laboratories at three regional tertiary care hospitals (Haukeland University Hospital (HUH), Oslo University Hospital (OUH) and Stavanger University Hospital (SUH)) from January 2015 until January 2016 were prospectively included in the study, provided there was enough material left after routine diagnostics to prepare a minimum of one cell smear or tissue section for the study (Fig. 1). Acellular fluid samples and all samples from patients that had received TB treatment during one year prior to the study, were excluded.

Because very few formalin-fixed biopsies from patients with presumptive TB had been prospectively included, a retrospective inclusion of biopsy specimens was also performed. At HUH and SUH, all samples included in the study from the microbiology laboratories were cross-checked with the pathology laboratory registers to see if the same sample, or a different sample material collected from the same location at the same time, had been sent to the pathology laboratories. At OUH, a list of all biopsies sent for TB diagnostics to the microbiology laboratory during 2015 was cross-checked with the pathology register to find the samples that had been sent for both departments. Based on these searches, formalin-fixed biopsies from the pathology departments were included if they showed any type of inflammation or necrosis. Biopsies with a histopathological diagnosis other than TB (e.g. malignancy) or no pathological findings were not included as these samples will not be subjected to TB specific tests at the pathology laboratory in a routine clinical setting.. Additionally, all formalin-fixed biopsies that had been subjected to a TB specific nested-PCR as part of routine diagnostics at Department of Pathology at HUH, were retrospectively included.

### Sample processing and routine TB diagnostic procedures

All samples were subjected to routine TB diagnostics at the inclusion hospitals according to local diagnostic algorithms. At the microbiology laboratories, FNAs in saline and fluid samples were used unconcentrated if volume < 10 mL and concentrated by centrifugation before resuspension of sediment in saline if the sample volume was > 10 mL. Biopsy specimens were mechanically homogenized and resuspended in saline. Cell smears were stained using the Ziehl-Neelsen or Auramine method for detection of AFB. For the study, a minimum of one cell smear from fluid samples was prepared on a Superfrost glass, air-dried for 20 min, fixed in absolute ethanol for 20 min and stored at room temperature. A standard NALC-NaOH decontamination procedure was performed on the remaining sample material if the sample was non-sterile, before appropriate sample volumes were seeded in liquid medium (BACTEC MGIT), and for most of the samples, also onto solid medium (Lowenstein-Jensen). At HUH, all lymph node specimens, sterile fluids and aspirates and most biopsies were cultured both before and after NALC-NaOH decontamination, and lymph node specimens were also cultured at 28 °C. If PCR was requested by the clinician, a 1-step PCR-based tests (1-step PCR) including Cobas Taqman MTB (Roche, Switzerland) at OUH, Abbott Real Time MTB (Abbott, United States) at SUH and Genotype MTBDR plus (Hain Lifescience, Germany) at HUH, was performed. All samples with a remaining volume of > 0.5 mL, were stored at − 80 °C for later analysis with Xpert Ultra.

At the pathology laboratories, biopsy specimens were routinely fixed in PBS buffered formalin and embedded in paraffin before tissue sections were prepared for histology. Fine-needle aspiration from lymph nodes was performed by local clinicians or pathologists and cell smears for cytology were directly prepared and fixed after sample collection. If microscopy for AFB was requested by the pathologist, the Ziehl-Neelsen (HUH, SUH), Auramine (OUS) or Fite Faraco [36] (OUS) method was used. Additionally, a previously developed in-house nested-PCR (n-PCR) for detection of the MTBC-specific *IS6110* sequence in DNA extracted from archived material [37] was also performed on the samples as part of routine diagnostics at HUH only, if requested by the pathologist.

### Xpert ultra

Xpert Ultra was performed on all the frozen sample material during the autumn 2018, except for pleural fluid samples, which were only subjected to Xpert Ultra if TB was mentioned as a differential diagnosis on the request form or in patient records. This was done to exclude clinically irrelevant samples, as many pleural fluid samples are routinely sent for TB diagnostics, even when the pre-test probability of TB is very low. Samples were thawed at room temperature and processed according to the manufacturer’s protocol. All but two samples (both volume 0.25 mL) had a sample volume of minimum 0.5 mL. Samples with volume < 0.7 mL (*n* = 20) were added sample reagent to sample in a 3:1 ratio, whereas a ratio of 2:1 was used for samples with a volume of 0.7 mL or more (*n* = 28).

### Immunostaining with MPT64

The MPT64 test was performed by a laboratory technician in parallel to routine TB diagnostics at Department of Pathology at HUH. The request form with clinical information, sometimes including results of TB diagnostic tests, was available to the technician. The test was performed using an in-house polyclonal rabbit anti-MPT64 antibody at 1:250 dilution together with the Dako Envision + System-HRP kit (Agilent, Santa Clara, CA), according to the manufacturer’s protocol with some modifications as earlier described [26, 28]. Briefly, tissue sections were deparaffinized with xylene, before tissue sections and cell smears were rehydrated through decreasing grades of alcohol. Microwave antigen retrieval in citrate buffer, pH 6.2, was then performed on tissue sections only. Further, tissue sections and cell smears were washed in distilled water for 10 min and incubated with hydrogen peroxide for 20 min. The primary anti-MPT64 antibody was applied and the slides were incubated for 60 min, before horse-radish conjugated secondary anti-rabbit antibody was applied for 45 min. Thereafter, the substrate (3-amino-9-ethylcarbazol) was added to the slides for 10 min for smears and 15 min for biopsies, followed by counterstaining with Mayer’s haematoxylin and mounting with Immu-Mount (Thermo Fisher Scientific, United States). Slides were washed with wash buffer (0.05 mol/L Tris/HCl buffered saline with 0.05% Tween 20, pH 7.6) between incubation steps.

### Evaluation of immunostaining

A laboratory technologist was trained to screen the MPT64 test stained cell smears prepared from fluid samples. Screening was performed at a total magnification of 200x and more detailed evaluation at 400x. Fluid samples screened possibly positive were examined by a designated pathologist, who also evaluated all biopsies and FNAs, according to a previously developed guideline for interpretation [35]. Briefly, a sample was positive if a minimum of two granular red-brown coloured spots, either observed intracytoplasmic in inflammatory cells or extracellularly in necrotic material, were present in the sample. If only one typical spot was present, or if the staining was not strongly granular, the test was evaluated as weakly positive. No staining, nuclear staining or extracellular granular staining in non-necrotic areas were interpreted as negative. Clinical information on the request form, which sometimes included information about results of routine TB diagnostics, was available to the pathologist.

### Categorisation of samples and patients according to a composite reference standard

A composite reference standard (CRS), including both microbiologically confirmed TB and clinically diagnosed TB, was used to define a TB case. Results of routine TB diagnostic tests and cyto/histopathological examination were obtained from the laboratory information systems. Medical records for all patients with culture and PCR negative samples were checked for a clinical TB diagnosis 8 months after the inclusion of samples had finished. According to the CRS, a patient was defined as a *confirmed TB case* if a culture and/or PCR (1-step PCR and/or n-PCR) positive sample was registered during the inclusion period or on a repeat sample until 8 months afterwards. Culture and/or PCR positive samples were classified as *confirmed samples from confirmed TB cases*, whereas culture and PCR negative samples from patients that were diagnosed with microbiologically confirmed TB within 8 months after end of inclusion, were classified as *unconfirmed samples from confirmed TB cases*. A patient that had been given a clinical TB diagnosis and successfully completed a full course of TB treatment, was defined as a *clinically diagnosed TB case*. Patients with culture and PCR negative samples that improved without treatment, or were given a diagnosis other than TB, or had a negative interferon-gamma-release assay, or had stable symptoms and negative results of TB diagnostics at repeated controls until 8 months after the inclusion had finished, were defined as *non-TB cases*. Patients that did not fit into any of these categories were classified as *uncategorised cases*.

### Statistical analysis

Test performance was evaluated using one sample per case. When multiple samples were included from one case, the first sample collected from non-TB cases and the first confirmed TB sample collected from TB cases was chosen for analysis. For TB cases with multiple unconfirmed samples included, the sample with cyto/histopathological findings most strongly suggestive of TB was chosen. Sensitivity, specificity and accuracy were calculated using the CRS as reference method.

## Results

### Clinical samples

A total of 465 samples received for TB diagnostics at the inclusion hospitals were consecutively sent to the HUH during the study period (Fig. 1). Additionally, 97 samples were retrospectively included from the same hospitals. After exclusion of 202 samples for various reasons, the remaining 360 samples from 288 patients were classified using the CRS. Twenty-six patients were confirmed TB cases, 5 clinically diagnosed TB cases, 243 non-TB cases and 14 uncategorised cases. Uncategorised cases were excluded, leaving samples from 274 patients for analysis. Clinical characteristics for the included samples are shown in Table 1. Pleural fluid was the most common sample type. The MPT64 test was performed on all samples, whereas the type and number of routine TB diagnostic tests performed on the samples varied. HIV status was unknown for the study participants.

**Table 1:** Characteristics of samples included (one sample per patient)

		TB cases^1^	non-TB cases
		*n* = 31	*n* = 243
Sample material			
Lymph node aspirates		7	4
Lymph node biopsies		7	17
Other biopsies		12	52
Pus		2	13
Pleural fluid		3	133
Ascites		0	8
Pericardial fluid		0	3
Synovial fluid		0	10
Other fluids		0	3
Number of samples per patient^2^			
1 sample		24	200
2 samples		6	33
3 samples		0	6
4 samples		1	3
5 samples		0	1
	Biopsies	Fine-needle aspirate	Puss and fluid samples
	*n* = 88	*n* = 11	*n* = 175
Sample sent to both microbiology and pathology laboratories	**68**	**9**	**83**
Microscopy^3^	59	9	43
1-step PCR	32	6	8
Nested-PCR	12	4	1
Culture	68	9	83
Sample only sent to the microbiology laboratory	**N/A**	**N/A**	**92**
Microscopy	N/A	N/A	51
1-step PCR	N/A	N/A	12
Culture	N/A	N/A	92
Sample only sent to the pathology laboratory	**20**	**2**	**N/A**
Microscopy	4	0	N/A
Nested-PCR	17	1	N/A

Abbreviations: TB, tuberculosis; PCR, polymerase chain reaction, N/A, not applicable

^1^Includes 5 clinically diagnosed patients

^2^Number of samples per patient included at different time points. Patients with the same material from the same site (*n* = 27), different material from the same site (*n* = 13), material from different locations (*n* = 6), material from different locations, multiple samples collected from some of these locations (*n* = 4)

^3^Microscopy performed at pathology and/or microbiology laboratory. A sample with discordant microscopy results between the laboratories (*n* = 2) is registered as positive

Among the 97 retrospectively included formalin-fixed biopsies, 13 biopsies were included because they had been subjected to a TB specific n-PCR at the pathology laboratory at HUH, due to histopathological findings suggestive of TB. These samples had not been sent for TB diagnostics at the microbiology laboratory and TB was not mentioned as a differential diagnosis on the request form. Four of 13 samples were n-PCR positive, and TB was thus, an unexpected finding in these cases.

### MPT64 test performance compared to routine TB diagnostics and Xpert ultra

#### Biopsy specimens

Using the CRS, the sensitivity (95% CI) of the MPT64 test in formalin-fixed biopsies was 37% (16–62), compared to 20% (4–48), 37% (16–62) and 50% (23–77) for microscopy, PCR-based tests pooled and culture respectively (Tables 2 and 3). Against PCR (1-step PCR and n-PCR pooled) as a reference standard, the sensitivity of the MPT64 test was 71% (5/7, 95% CI 29–96). However, in PCR negative, culture positive biopsies (*n* = 6), the MPT64 test was negative in all samples. One of the 69 non-TB biopsies was MPT64 test positive, yielding a positive predicitive value of 88% (7/8 MPT64 test positive biopsies were from TB cases) and an excellent specificity of 99% (95% CI 92–100). Granulomatous inflammation with necrosis, the most specific histopathological finding suggestive of TB, was present in 13/19 biopsies from TB cases and 13/69 non-TB biopsies (Table 4). This gives histopathology a sensitivity, specificity and positive predicitive value of 68% (43–87), 81% (70–90) and 50% (36–64) respectively against the CRS. Among biopsies from non-TB cases, 5 samples were bacteriologically confirmed NTM infections and another 3 samples came from patients with a probable, though not confirmed, NTM infection. The MPT64 test was negative inn all these samples.

**Table 2:** Results of routine TB diagnostic tests, Xpert Ultra and the MPT64 test performed on samples

		Positive samples/total samples (%)					
	Number of cases	Micro-scopy^a^	Culture	1-step PCR	Nested-PCR	Xpert Ultra	MPT64 test
TB cases							
All samples	31	6/27 (22)	16/26 (62)	6/18 (33)	7/11 (64)	4/7 (57)	10/31 (32)
Lymph node biopsies	7	1/6 (17)	2/6 (33)	0/5 (0)	2/2 (100)	N/A	3/7 (43)
Other biopsies	12	2/9 (22)	5/8 (63)	1/7 (14)	4/6 (67)	N/A	4/12 (33)
Pus samples	2	0/2 (0)	1/2 (50)	1/1 (100)	N/A	1/2 (50)	1/2 (50)
Lymph node aspirates	7	3/7 (43)	5/7 (71)	4/4 (100)	1/3 (33)	3/4 (75)	2/7 (29)
Fluid samples	3	0/3 (0)	3/3 (100)	0/1 (0)	N/A	0/1 (0)	0/3 (0)
Confirmed TB case -confirmed sample	22^b^	5/19 (26)	16/17 (94)	6/13 (46)	7/9 (78)	4/5 (80)	8/22 (36)
Culture positive sample	16	4/16 (25)	16/16 (100)	5/12 (42)	2/4 (50)	4/5 (80)	3/16 (19)
Culture positive, PCR positive sample	6	3/6 (50)	6/6 (100)	5/5 (100)	2/2 (100)	3/3 (100)	3/6 (50)
Culture positive, PCR negative sample	8	1/8 (13)	8/8 (100)	0/7 (0)	0/2 (0)	1/2 (50)	0/8 (0)
1-step PCR positive sample	6	4/6 (67)	5/6 (83)	6/6 (100)	1/1 (100)	3/3 (100)	4/6 (67)
Nested-PCR positive sample	7	1/4 (25)	2/2 (100)	1/1 (100)	7/7 (100)	1/1 (100)	5/7 (71)
Confirmed TB case -unconfirmed sample	4	0/3 (0)	0/4 (0)	0/3 (0)	N/A	N/A	2/4 (50)
Clinically diagnosed TB case	5	1/5 (20)	0/5 (0)	0/2 (0)	0/2 (0)	0/2 (0)	0/5 (0)
Non-TB cases							
All samples	243	7/139 (5)	0/226 (0)	0/40 (0)	0/24 (0)	1/41 (2)	39/243 (16)
Lymph node biopsies	17^c^	1/13 (8)	0/14 (0)	0/4 (0)	0/4 (0)	0/2 (0)	0/17 (0)
Other biopsies	52^d^	2/35 (6)	0/40 (0)	0/16 (0)	0/17 (0)	1/8 (13)	1/52 (2)
Pus samples	13^e^	3/13 (23)	0/13 (0)	0/4 (0)	0/1 (0)	0/8 (0)	4/13 (31)
Lymph node aspirates	4^f^	1/2 (50)	0/2 (0)	0/2 (0)	0/2 (0)	0/1 (0)	2/4 (50)
Fluid samples	157	0/76 (0)	0/157 (0)	0/14 (0)	N/A	0/22 (0)	32/157 (20)

Abbreviations: TB, tuberculosis; PCR, polymerase chain reaction

^a^Microscopy performed at microbiology laboratory and/or pathology laboratory. A sample with discordant microscopy results between the laboratories (n = 2) is registered as microscopy positive

^b^One of the nested-PCR positive patients was not started on TB treatment, and only followed by controls

^c^6 cases of NTM infection (2 NTM culture positive, 1 NTM specific PCR positive, 3 given a clinical diagnosis)

^d^2 cases of NTM infection (2 NTM specific PCR positive)

^e^3 cases of NTM infection (3 microscopy and NTM culture positive)

^f^1 case of NTM infection (microscopy, NTM culture and NTM specific PCR positive. This case was also MPT64 positive)

**Table 3:** Test accuracy for various routine diagnostic tests, Xpert Ultra and the MPT64 test using a composite reference standard

	Test performed on number of samples	Sensitivity % (95% CI)	Specificity % (95% CI)	Overall accuracy %
All samples (*n* = 274)				
Microscopy	166	22 (9–42)	95 (90–98)	83
Culture	252	62 (41–80)	100 (98–100)	96
PCR (1-step PCR and n-PCR pooled)	87	44 (26–65)	100 (94–100)	83
Xpert Ultra	48	57 (18–90)	98 (87–100)	92
MPT64 test	274	32 (17–51)	84 (79–88)	78
Lymph node biopsies (n = 24)				
Microscopy	19	17 (0–64)	92 (64–100)	68
Culture	20	33 (4–78)	100 (77–100)	80
PCR (1-step PCR and n-PCR pooled)	15	29 (4–71)	100 (63–100)	67
Xpert Ultra	2	N/A	100 (16–100)	–
MPT64 test	24	43 (10–82)	100 (80–100)	83
All biopsies (n = 88)				
Microscopy	63	20 (4–48)	94 (83–99)	76
Culture	68	50 (23–77)	100 (93–100)	90
PCR (1-step PCR and n-PCR pooled)	57	37 (16–62)	100 (91–100)	79
Xpert Ultra	10	N/A	90 (56–100)	–
MPT64 test	88	37 (16–62)	99 (92–100)	85
Lymph node aspirates (n = 11)				
Microscopy	9	43 (10–82)	50 (1–99)	44
Culture	9	71 (29–96)	100 (16–100)	78
PCR (1-step PCR and n-PCR pooled)	9	67 (22–96)	100 (29–100)	78
Xpert Ultra	5	75 (19–99)	100 (3–100)	80
MPT64 test	11	29 (4–71)	50 (7–93)	36
Pus samples (*n* = 15)				
Microscopy	15	0 (0–84)	77 (46–95)	67
Culture	15	50 (1–99)	100 (75–100)	93
PCR (1-step PCR and n-PCR pooled)	6	100 (3–100)	100 (48–100)	100
Xpert Ultra	10	50 (1–99)	100 (63–100)	90
MPT64 test	15	50 (1–99)	69 (39–91)	67
Fluid samples (*n* = 160)				
Microscopy	79	0 (0–71)	100 (95–100)	96
Culture	160	100 (29–100)	100 (98–100)	100
PCR (1-step PCR and n-PCR pooled)	15	0 (0–98)	100 (77–100)	93
Xpert Ultra	23	0 (0–98)	100 (85–100)	96
MPT64 test	160	0 (0–71)	80 (72–86)	78

Abbreviations: PCR, polymerase chain reaction; CI, confidence interval

**Table 4:** Cyto/histopathological findings in biopsy and fine-needle aspirate samples

	TB cases				Non-TB cases
	Confirmed sample	Unconfirmed sample	Clinically diagnosed sample	Total TB cases	
Histomorphology, biopsies (n = 88)	n = 13	n = 3	n = 3	*n* = 19	*n* = 69
Granulomatous inflammation with necrosis	9	3	1	13	13
Granulomatous inflammation without necrosis	1		2	3	13
Subacute or chronic inflammation and necrosis	3			3	4
Abundant necrosis					3
Subacute or chronic inflammation					22
Acute inflammation					3
Malignant tumor					4
Benign tumor					2
Fibrosis					1
Foreign body granuloma					1
Lymph node hyperplasia					1
Mesothelial proliferation					1
No pathology					1
Cytomorphology, FNAs (n = 11)	n = 5	n = 1	n = 1	*n* = 7	n = 4
Granulomatous inflammation with necrosis			1	1	1
Abundant necrosis	3			3	1
Fluid background, mostly RBCs	1			1	1
Subacute or chronic inflammation	1			1	1
Acute inflammation		1		1	

Abbreviations: TB, tuberculosis; FNA, fine-needle aspirate

#### Fine needle-aspirates and fluid samples

Abundant non-specific staining was observed in the cell smears prepared from FNAs, pus and fluid samples, and the MPT64 test performance was lower in these materials compared to the biopsies. Using the CRS, the sensitivity and specificity of the MPT64 test in lymph node FNAs was 29% (95% CI 4–71) and 50% (95% CI 7–93) respectively. Cytopathological findings suggestive of TB had low sensitivity and specificity for diagnosing TB. In pus and fluid samples, the sensitivity of all tests methods was difficult to evaluate due to few TB cases. All three pleural fluids from TB cases were culture positive and negative with all other tests. Two pus samples from TB cases were included. One was microscopy negative and positive with culture, 1-step PCR, Xpert Ultra and the MPT64 test, whereas the other sample was negative with all tests. Many non-TB pus and fluid samples were interpreted as weakly positive (*n* = 33) or positive (n = 3), and the specificity of the MPT64 test was 80% (95% CI 72–86) and 69% (95% CI 39–91) in fluid and pus samples, respectively.

### Head-to-head comparison of various diagnostic methods

As the number of TB diagnostic tests performed on the samples varied greatly, the diagnostic performance of the different tests was also evaluated based on head-to-head comparisons (Table 5). There was no difference in the overall test performance between microscopy and the MPT64 test, which both detected the same number of TB cases as 1-step PCR, and fewer TB cases than n-PCR, Xpert Ultra and culture. Further, the subgroup comparisons of culture, 1-step PCR, microscopy and the MPT64 test showed that some samples were positive in one test and negative in the other and vice versa. The MPT64 test was positive in 4 microscopy negative samples, 2 1-step PCR negative samples and 3 culture negative samples, indicating added value of combining various TB diagnostic tests.

**Table 5:** Head-to-head comparison of different TB diagnostic tests, including Xpert Ultra and the MPT64 test among TB cases^1^

	All TB cases n = 31									
	MPT64 test performed		Microscopy performed		1-step PCR performed		Nested-PCR performed		Culture performed	
	n = 31		n = 27		*n* = 18		n = 11		n = 26	
	MPT64 test +	MPT64 test -	Micro-scopy +	Micro-scopy -	1-step PCR+	1-step PCR-	Nested-PCR+	Nested-PCR-	Culture +	Culture -
	*n* = 10	*n* = 21	n = 6	n = 21	n = 6	*n* = 12	n = 7	n = 4	n = 16	n = 10
Microscopy +	2	4								
Microscopy -	4	17								
1-step PCR +	4	2	4	2						
1-step PCR -	2	10	2	9						
Nested-PCR +	5	2	1	3	1	0				
Nested-PCR -	0	4	0	4	0	1				
Culture +	3	13	4	12	5	7	2	2		
Culture -	3	7	2	7	1	5	0	2		
Xpert Ultra +	2	2	2	2	3	0	1	1	4	0
Xpert Ultra -	0	3	0	3	0	1	0	1	1	2

Abbreviations: TB, tuberculosis; PCR, polymerase chain reaction

^1^TB cases include both microbiologically confirmed TB cases (n = 26) and clinically diagnosed TB cases (n = 5)

## Discussion

This is the first study in which the MPT64 test, an immunochemistry-based test for diagnosing EPTB, has been implemented in parallel to routine TB diagnostics in a low TB prevalence country with a high-resource health care system. Using a CRS that included clinically diagnosed TB cases, the test had a sensitvity (95% CI) of 37% (16–62) in formalin-fixed biopsies, compared to 37% (16–62) and 50% (23–77) for PCR-based tests pooled and culture, respectively. The specificity of the test was excellent (99, 95% CI 92–100) in formalin-fixed biopsies. In cell smears prepared from FNAs, pus and fluid samples, the test performance was low. Culture was found to be the most sensitive method for diagnosing TB in the study, with a disadvantage of long turnaround time. The study revealed that in this low TB incidence setting, many EPTB cases are incidentally detected based on histopathological findings in biopsy specimens that have not been sent for culture in parallel. Histopathological findings alone cannot confirm a TB diagnosis, and in these cases, the MPT64 test can supplement other rapid tests, including microscopy and n-PCR. This test is less prone to contamination than PCR and, in contrast to microscopy, can differentiate between MTBC and NTM infections. Thus, the MPT64 test may strengthen the TB diagnosis in a pathology laboratory in the absence of culture confirmation.

The MPT64 test performance was lower in the present study compared to previous studies [28–30, 35]. Against a CRS, the overall sensitivity was 32% (95% CI 17–51) for the MPT64 test, compared to 67–100% in previous studies [28–30, 35]. The use of different composite reference standards and variable TB prevalence across the studies may contribute to this variation. All previous studies were conducted in high TB burden settings, in which a higher pre-test probability of TB combined with potentially more advanced stage of TB disease at the time of diagnosis, may lead to higher test sensitivity. Still, also when using culture as a reference standard, the overall MPT64 test sensitivity was lower (19, 95% CI 4–46) compared to previous studies (75–100%) [26, 28, 30, 35, 37]. This could partly be explained by different procedures for culture used across the studies. Apportioning of smaller sample volumes for culture and long transportation time to the TB laboratory, potentially reducing the viability of the bacilli, may have reduced the sensitivity of culture in previous studies [35]. In most of the previous studies, all samples were decontaminated and seeded onto only 1 tube of solid medium, whereas 2–8 culture tubes per sample were used for most samples in the present study, including culturing of material not treated with NALC-NaOH for many samples. These factors can lead to increased sensitivity of culture in our study, especially in paucibacillary specimens with non-uniform distribution of bacilli. Further, the use of different reference standards makes it challenging to compare the studies. For validation of the MPT64 test, n-PCR has been used as a reference standard in most previous studies, yielding a sensitivity of 72–100% [26–29, 31, 35]. In the present study, n-PCR was only performed on a subgroup of samples and could not be used for validation alone. However, when using n-PCR as a reference standard in this subgroup, the sensitivity of the MPT64 test was 71% (95% CI 29–96). This is close to previous findings. Moreover, all culture positive samples were n-PCR positive in previous studies, whereas the present study included several culture positive, but PCR negative samples (*n* = 8). The MPT64 test was negative in all these culture positive, PCR negative samples. Assuming that culture positive, PCR negative samples have a lower bacterial load than culture positive, PCR positive samples, these results indicate that the MPT64 test is not sensitive enough to detect samples with very low bacterial load. However, the long turnout time of culture does not help clinicians to make a timely diagnosis. Further, although culture performed under optimal conditions is the most sensitive method for diagnosing EPTB in the present study, TB culture facilities are not available in most TB endemic areas, in which TB diagnostics are most needed.

The specificity of the MPT64 test in biopsy specimens was very high and comparable to results observed in previous studies, whereas the specificity in cell smears prepared from FNAs, pus and fluid samples was lower. In lymph node FNAs, the specificity was only 50%, However, this was based on only two MPT64 test positive non-TB cases of a total of four non-TB cases, which gives low power to the estimate. In pus and fluid samples, non-specific false positive staining was observed in a large proprotion of the smears and made interpretation challenging. The non-specific staining may have been caused by suboptimal sample handling at the microbiology laboratories where samples could be stored cold for more than one day before preparation of smears, as indicated by cell lysis in many samples. Long storage time may have affected the antigen integrity and increased non-specific binding. In contrast, smears in previous studies were prepared immediately after sample collection.

The low specificity of the test in cell smears has a greater impact in this low prevalence setting compared to a high prevalence setting because more false positive cases and unecessary treatment must be accepted for every true positive case detected. Thus, the results of the present study indicate that the MPT64 test is not useful for diagnosis of EPTB in cell smears. In biopsy specimens, on the other hand, the test was highly specific. It was negative in clinically relevant non-TB samples with various types of inflammation and in all samples from patients with NTM infections. NTM infection is an important differential diagnosis to EPTB, as 31% of the microbiologically confirmed mycobacterial infections were NTM in the present study.

There are limitations to the study. The low number of TB cases gives low power to sensitivity estimates.. Further, the exlusion of culture and PCR negative samples because information about clinical TB diagnosis was not available (Fig. 1), in addition to the exclusion of biopsies with a histopathological diagnosis other than TB or no pathological findings, leads to a selection bias in favour of samples with a higher pre-test probability of TB, which could affect specificity estimates. As the study was designed to evaluate the MPT64 test performance in a routine setting, we did not intervene in sample handling or TB diagnostic algorithms at the inclusion sites, leading to many suboptimally prepared samples for the MPT64 test. Samples from patients with presumptive EPTB were often not sent for TB diagnostics both to microbiology and pathology laboratories, as would have been expected according to good clinical practice. Not only may this lead to diagnostic delay since the available diagnostic tools are not fully utilized in difficult-to-diagnose cases, but it also makes it difficult to compare test performance in the present study because the types and number of tests performed per sample varied greatly. Hence, more controlled validation studies with larger cohorts are needed to fully asses MPT64 test performance in a low TB incidence high-resource setting.

## Conclusions

The diagnosis of EPTB is challenging in a high-resource, low-TB incidence country. The awareness of TB is often low and routine TB diagnostic tests are not able to identify all EPTB cases. The MPT64 antigen detection test has a good positive predictive value and an excellent specificity in formalin-fixed biopsies and is implementable in pathology laboratories. In the absence of culture, the MPT64 test may contribute to strengthen the TB diagnosis in formalin-fixed biopsies when used in combination with microscopy and PCR-based tests, and thus, has an added value in TB diagnostics in this setting.

## Data Availability

The datasets generated and analysed during the current study are available from the corresponding author on reasonable request.

## Abbreviations

AFB: Acid fast bacilli
CRS: Composite reference standard
EPTB: Extrapulmonary tuberculosis
FNA: Fine needle aspirate
HUH: Haukeland University Hospital
MTBC: *Mycobacterium tuberculosis* complex
n-PCR: Nested-PCR
NTM: Non-tuberculosis mycobacteria
OUH: Oslo University Hospital
SUH: Stavanger University Hospital
TB: Tuberculosis
WHO: World Health Organisation
